# A Bayesian network-based predictive model for gout onset risk: associations with traditional Chinese medicine constitution in hyperuricemic populations

**DOI:** 10.3389/fmed.2026.1868361

**Published:** 2026-09-03

**Authors:** Xiaobing Tian, Junlong Chen, Qianqiong Chen, Runxia Teng

**Affiliations:** 1 Rheumatology Department, Shanghai General Hospital Jiuquan Hospital (People's Hospital of Jiuquan), Jiuquan, China; 2 Traditional Chinese Medicine Department, Shanghai General Hospital Jiuquan Hospital (People's Hospital of Jiuquan), Jiuquan, China

**Keywords:** Bayesian network, Chinese medicine constitution, hyperuricemia gout traditional, predictive model, risk of illness

## Abstract

**Objective:**

To develop a Bayesian network model for predicting gout onset risk in hyperuricemic individuals based on TCM body constitutions, quantify their influence, and provide evidence for early warning and TCM intervention.

**Method:**

A total of 826 hyperuricemia (HUA) patients were enrolled in this prospective cohort study. All participants underwent standardized TCM constitution identification per “Classification and Identification of Constitution in Traditional Chinese Medicine.” Clinical variables including gender, age, BMI, serum uric acid, and triglycerides were collected. A Bayesian network was constructed via the Hill-Climbing algorithm, and its performance was validated by 10-fold cross-validation.

**Result:**

During follow-up, 217 participants progressed to gout (incidence 26.27%). Phlegm-dampness (24.1%) and damp-heat (21.8%) were the most prevalent TCM constitutions. Their prevalence was significantly higher in the gout group (38.7% and 32.7%) than in the non-gout group (*P* < 0.001). Bayesian network analysis identified serum uric acid, BMI, phlegm-dampness, and damp-heat constitutions as direct predictors of gout onset. Conditional probabilities: phlegm-dampness 62.3%, damp-heat 54.1%, balanced 8.4%. When phlegm-dampness coexisted with serum uric acid ≥540 μmol/L, gout incidence rose to 81.6%. The model achieved favorable efficacy: accuracy = 0.832, AUC = 0.847, sensitivity = 0.811, specificity = 0.843.

**Conclusion:**

Phlegm-dampness and damp-heat constitutions are key predisposing TCM patterns for gout in hyperuricemic individuals. The Bayesian network model effectively quantifies probabilistic links between TCM constitutions and gout risk, offering a reliable tool for personalized risk stratification and TCM-guided preventive management.

## 1 Introduction

Hyperuricemia (HUA) is a metabolic disturbance triggered by abnormal purine metabolism or impaired uric acid excretion in the human body. Its core feature is that the fasting blood uric acid level exceeds the normal threshold (1). As a key biochemical foundation for gout pathogenesis, hyperuricemia (HUA) is closely associated with gout development. Epidemiological data indicate that the prevalence of HUA among Chinese adults has reached 14.0%, while the incidence of gout is 11.1 per 100 person-years. Both conditions exhibit a consistent annual upward trend, with a progressively younger age at onset, posing a substantial threat to national public health (2–4). Acute gout flares typically present with joint erythema, swelling, warmth, and pain. Repeated attacks can lead to joint deformity and functional disorders. In severe cases, it can affect the kidneys, causing serious complications such as uric acid nephropathy and renal failure (5, 6). At present, modern medicine's prediction of the risk of gout mainly focuses on objective indicators such as blood uric acid levels, gender, age, body mass index, and lipid metabolism, and uses traditional statistical methods such as Logistic regression and Cox proportional hazards regression to construct predictive models (7–9).

The theory of constitution in traditional Chinese medicine, as a core component of TCM theory, emphasizes that constitution determines disease susceptibility and holds that individual constitution differences are the key internal factors for the occurrence, development and outcome of diseases (10). Within the domain of traditional Chinese medicine, HUA and gout are mostly classified under the categories of turbidity arthralgia, dampness arthralgia, and phlegm-stasis arthralgia. The core pathogenesis of their onset is closely related to dampness, heat, phlegm and blood stasis (11). Clinical investigations have verified that individuals with hyperuricemia (HUA) and gout commonly exhibit deviated constitutions, among which phlegm-dampness and damp-heat patterns represent high-risk predispositions (12). Nevertheless, existing investigations into the link between traditional Chinese medicine constitution and gout susceptibility among hyperuricemic individuals remain largely confined to descriptive assessments, with insufficient quantitative evaluation of the correlative strength between these factors. It remains challenging to accurately define the predictive weight of distinct constitutional patterns on gout onset, thereby failing to offer targeted evidence for personalized clinical strategies.

Bayesian Network (Bayesian Network, BN) is an uncertain reasoning model based on probability theory and Graph theory, which visually expresses the causal relationship and conditional probability dependency relationship between variables in the form of a Directed Acyclic Graph (DAG). It has powerful advantages in multi-factor interaction analysis, uncertainty reasoning and interpretability (13). This algorithm does not need to assume the linear relationship between variables and can effectively handle multi-factor, non-linear and uncertain data in medical research. It has been extensively utilized in medical domains including disease risk projection and pathogenic factor investigation (14).

Based on this, this study takes traditional Chinese medicine constitution as the core variable, combines general data and laboratory indicators, and uses the Bayesian network algorithm to construct a gout risk prediction model for the HUA population, quantifying the association strength between traditional Chinese medicine constitution and gout incidence, and clarifying the influence probability of different constitution types on gout incidence. It aims to provide scientific basis and practical tools for the early warning, hierarchical management and individualized constitution adjustment of gout in traditional Chinese medicine.

## 2 Objects and methods

Informed consent was obtained from participants prior to their involvement in the study. The participants were fully informed of the study's purpose, potential risks, and anticipated benefits before providing their informed consent. For any participants under the age of 16, additional informed consent was obtained from their parents or legally authorized representatives.

### 2.1 Research object

A total of 826 individuals diagnosed with hyperuricemia (HUA) were enrolled as study participants, recruited from the physical examination center and rheumatology and immunology outpatient clinics between December 2021 and December 2024. All participants fulfilled the hyperuricemia diagnostic criteria outlined in the *Chinese Guidelines for the Diagnosis and Management of Hyperuricemia and Gout (2019)* (15). The data lock-up date for this study is December 31, 2025.

#### 2.1.1 Inclusion criteria

(1) Fulfilling the established diagnostic standards for hyperuricemia, with fasting serum uric acid concentrations exceeding 420 μmol/L in males and 360 μmol/L in females;

(2) Aged within the range of 18 to 75 years old;

(3) Voluntarily enrolled in the present study and provided written informed consent;

(4) Able to fully cooperate with TCM constitution assessment, laboratory examinations, and scheduled follow-up visits;

(5) Participants completed a follow-up duration of at least 12 months and possessed full clinical documentation.

#### 2.1.2 Exclusion criteria

(1) Patients with secondary hyperuricemia (HUA), including drug-induced and disease-related HUA;

(2) Those who have been clearly diagnosed with gout or have a history of acute gout attacks;

(3) Those with severe organic diseases;

(4) Pregnant or lactating women;

(5) Individuals with psychiatric conditions or cognitive deficits who cannot comply with study procedures;

(6) Researchers with incomplete clinical data, those who lost contact during follow-up, or those who withdrew halfway.

#### 2.1.3 Grouping method

All participants received prospective follow-up. The initial episode of acute gout during surveillance was defined as the primary endpoint, and individuals were stratified into the gout and non-gout cohorts according to endpoint occurrence.

The diagnosis of gout meets the classification criteria of gout (16, 17): (1) Acute arthritis attack ≥1 time; (2) Urate crystals were detected in the joint fluid. (3) Monosodium urate crystal deposition (tophi); (4) Hyperuricemia; (5) Asymmetric joint swelling; (6) Red joints; (7) Pain or swelling in the first metatarsophalangeal joint; (8) Episode involving the unilateral first metatarsophalangeal joint; (9) Episode involving the unilateral tarsal joint; (10) Presence of suspected tophi; (11) Elevated erythrocyte sedimentation rate (ESR) during arthritis flare; (12) Asymmetric intra-articular swelling on radiography; (13) Subcortical cysts without bone erosion on radiography. A definitive diagnosis is established when at least four criteria are satisfied and other arthritic disorders are ruled out.

### 2.2 Research methods

#### 2.2.1 General information collection

A uniformly designed questionnaire was adopted. Researchers who had received systematic training conducted face-to-face interviews with the research subjects to collect general clinical data, including gender, age, height, weight, smoking history, drinking history, and past medical history (hypertension, diabetes, hyperlipidemia, etc.), calculate the body mass Index (BMI) [see Equation (1)].

$$\begin{array}{l} BMI\;=\;\frac{Weight\left(kg\right)}{Height^{2}\left(m^{2}\right)} \end{array}$$ (1)

According to the classification standard of BMI for Chinese adults, they are divided into underweight (BMI < 18.5 kg/m^2^), normal (18.5–23.9 kg/m^2^), overweight (24.0–27.9 kg/m^2^), and obese (BMI ≥ 28.0 kg/m^2^) (18).

#### 2.2.2 Laboratory index testing

Fasting morning venous blood (5 mL) was obtained from all participants via antecubital venipuncture and collected in vacuum tubes. After centrifugation at 3,000 r/min with a 10-cm radius for 10 min, serum specimens were isolated. An automated biochemical analyzer was used to measure key laboratory parameters, including serum uric acid (SUA), triglycerides (TG), total cholesterol (TC), fasting blood glucose (FBG), creatinine (Cr), and blood urea nitrogen (BUN). All determinations were performed in strict accordance with standardized laboratory protocols to guarantee the validity and reproducibility of analytical outcomes.

#### 2.2.3 TCM constitution identification

All participants were classified by two licensed TCM clinicians with attending or higher qualifications and no less than 5 years of clinical practice, following the *Classification and Determination of TCM Constitutions* [ZYYXH/T 157-2009 (19)] This guideline categorizes TCM constitutions into nine distinct categories: balanced, qi-deficiency, Yang-deficiency, Yin-deficiency, phlegm-dampness, damp-heat, blood-stasis, qi-stagnation, and special diathesis. A standardized 60-item inventory was used for quantitative assessment, with each item rated on a 1–5 Likert scale. Profiles were assigned according to aggregate and subscale scores. During the process of constitution identification, when two traditional Chinese medicine practitioners have different opinions, a third senior traditional Chinese medicine practitioner will conduct a recheck and determination to ensure the consistency of the constitution identification results (Kappa value = 0.87, *P* < 0.001), which meets the requirements of clinical research.

#### 2.2.4 Follow-up plan

A combined approach of telephone follow-up and outpatient follow-up was adopted for the prospective follow-up. The follow-up began on the day the research subjects were included in the study. The follow-up endpoint was defined as the first occurrence of acute gout attack by the research subjects, or after 36 months of follow-up, or until the data lock of this study on December 31, 2025 (considered as the end of follow-up). The follow-up frequency was set as follows: 1 follow-up per month in the first 6 months after enrollment, and 1 follow-up every 3 months thereafter.

During each follow-up, the health status of the research subjects is fully recorded, with a particular focus on verifying whether there is an acute attack of gout, the specific time of the attack, the location of the attack, and related clinical symptoms; at the same time, the daily diet, exercise and other lifestyle habits of the subjects, as well as the situation of lowering uric acid and other concurrent medications, are also recorded. All clinical data are updated and improved in a timely manner. For the subjects who lost contact during the follow-up period, continuous tracking is conducted through multiple channels such as family members and reserved emergency contacts. If the subjects are continuously out of contact for more than 3 months, they will be determined as lost to follow-up and excluded. This study ultimately included 826 subjects with hyperuricemia, all of whom completed the follow-up, and there were no cases of loss to follow-up. The follow-up success rate was 100%.

The median follow-up duration in this cohort was 28.7 months, with an average follow-up duration of 26.4 months. The time span from the subject's enrollment was from December 2021 to December 2024. The last subject was enrolled on December 2024, and by the data lock date on December 31, 2025, the last enrolled subject had met the minimum follow-up duration of 12 months. The follow-up duration of all subjects met the inclusion criteria. The longest follow-up duration for the early enrolled subjects was 36 months, which was in line with the preset follow-up design.

#### 2.2.5 Construction of Bayesian network model

##### 2.2.5.1 Variable filtering and definition

Based on relevant domestic and international research and clinical practice, the related variables influencing the incidence of gout in the HUA population were screened. A total of 10 node variables were included, which were divided into basic variables, core variables and outcome variables:

Basic variable Gender (male = 1, female = 0), age (< 40 years old = 0, 40–59 years old = 1, ≥60 years old = 2), BMI (normal = 0, overweight = 1, obese = 2), SUA (< 480 μmol/L = 0, 480–540 μmol/L = 1 >540 μmol/L = 2), TG (< 1.7 mmol/L = 0, ≥1.7 mmol/L = 1), TC (< 5.2 mmol/L = 0, ≥5.2 mmol/L = 1), FBG (< 6.1 mmol/L = 0, ≥6.1 mmol/L = 1);

Core variables: Traditional Chinese Medicine Constitution (balanced constitution = 0, Qi deficiency constitution = 1, Yang deficiency constitution = 2, Yin deficiency constitution = 3, phlegm-dampness constitution = 4, damp-heat constitution = 5, blood stasis constitution = 6, qi stagnation constitution = 7, special endowment constitution = 8);

Outcome variable: gout onset (no onset = 0, onset = 1).

##### 2.2.5.2 Structural learning

The Hill-Climbing (HC) algorithm is adopted for Bayesian network structure learning. This algorithm iteratively optimizes directed connections among nodes and adopts the Bayesian Information Criterion (BIC) as the metric for structural assessment. The smaller the BIC value, It indicates that the better the fit between the network structure and the data. During the structural learning process, the maximum number of parent nodes is set to 3, and the number of iterations is 1,000 times. Through multiple iterations and optimizations, the optimal Bayesian network structure is finally determined.

##### 2.2.5.3 Parameter learning

After the structure is determined, Maximum Likelihood Estimation (MLE) is adopted for parameter learning. MLE estimates the conditional probabilities of each node variable under different states of its parent node by maximizing the likelihood function of the sample data. Construct a Conditional Probability Table (CPT) to quantify the probability dependencies among nodes. During the parameter learning process, Laplacian smoothing processing is adopted to avoid extreme cases with a probability of 0, ensuring the stability and reliability of parameter estimation.

##### 2.2.5.4 Model building tools

GeNIe 3.0 (BayesFusion, LLC., Pittsburgh, USA) was employed for Bayesian network establishment, structural inference, and parameter estimation, and the R 4.2.1 (R Foundation for Statistical Computing, Vienna, Austria) software was used for data preprocessing, structure optimization and model verification.

#### 2.2.6 Model performance evaluation

Performance of the established Bayesian network predictive model was assessed via 10-fold cross-validation. The 826 study participants were randomly allocated into 10 subsets: nine were employed as the training cohort for framework establishment, and one served as the validation subset for reliability testing. This iterative procedure was conducted 10 times, with the mean value across all rounds adopted as the final performance metric.

Four key indices were selected to quantify predictive efficacy: accuracy, area under the receiver operating characteristic curve (AUC), sensitivity, and specificity.

Accuracy represents the ratio of correctly classified samples to the total cohort, reflecting the global predictive capacity of the framework.

AUC quantifies the capacity to differentiate between affected and unaffected individuals; values closer to 1 denote stronger discriminative power, with scores exceeding 0.8 indicating favorable predictive performance.

Sensitivity is defined as the ratio of accurately identified cases to the total number of affected individuals, representing the framework's case-detection efficacy.

Specificity refers to the proportion of correctly recognized non-cases relative to all unaffected participants, illustrating the framework's ability to exclude disease-free individuals.

#### 2.2.7 Probabilistic reasoning analysis

Based on the constructed Bayesian network model, by setting evidence nodes, reasoning the posterior probability of outcome variables, analyzing the influence of different TCM constitution types and different laboratory index levels on the risk of gout onset in the HUA population, and clarifying the intensity of the core risk factors.

#### 2.2.8 Statistical methods

Statistical processing was performed with SPSS 26.0. Continuous variables were presented as mean±standard deviation ($\bar{x}$ ± *s*) and compared using independent-sample *t*-tests. Categorical variables were reported as case numbers and percentages [*n* (%)], with intergroup contrasts assessed by the χ^2^ test. Ordinal data were analyzed via rank-sum testing. Statistical significance was defined as *P* < 0.05.

## 3 Results

### 3.1 Comparison of baseline data of the research subjects

Among the 826 hyperuricemic patients, 217 were assigned to the gout group, consisting of 158 men (72.8%) and 59 women (27.2%), with a mean age of (52.3 ± 10.5) years. The non-gout group included 609 individuals, comprising 387 men (63.5%) and 222 women (36.5%), with a mean age of (48.6±11.2) years.

Baseline comparisons revealed that the male proportion, age, BMI, SUA, and TG concentrations in the gout group were significantly elevated compared with the non-gout group, with statistically significant differences (*P* < 0.05). No significant intergroup differences were detected in TC, FBG, Cr, BUN levels, or the prevalence of smoking and comorbidities (*P* > 0.05) (Table 1).

**Table 1:** Comparison of baseline data of the research subjects.

Index	Gout group (*n* = 217)	Non-gout group (*n* = 609)	*t*/χ^2^	*P*
Gender [*n* (%)]	–	–	–	–
Male	158 (72.8)	387 (63.5)	6.892	0.009
Female	59 (27.2)	222 (36.5)		
Age (years, $\bar{x}$ ±*s*)	52.3 ± 10.5	48.6 ± 11.2	3.985	< 0.001
BMI (kg/m^2^, $\bar{x}$ ±*s*)	27.8 ± 3.2	25.6 ± 3.5	7.542	< 0.001
SUA (μmol/L, $\bar{x}$ ±*s*)	532.6 ± 78.5	478.3 ± 82.1	8.216	< 0.001
TG (mmol/L, $\bar{x}$ ±*s*)	2.8 ± 1.1	2.1 ± 1.0	7.934	< 0.001
TC (mmol/L, $\bar{x}$ ±*s*)	5.6 ± 1.3	5.4 ± 1.2	1.872	0.061
FBG (mmol/L, $\bar{x}$ ±*s*)	6.3 ± 1.4	6.1 ± 1.3	1.568	0.117
Cr (μmol/L, $\bar{x}$ ±*s*)	88.5 ± 21.3	85.7 ± 20.8	1.429	0.153
Smoking history [*n* (%)]	89 (41.0)	235 (38.6)	0.387	0.534
Drinking history [*n* (%)]	102 (47.0)	248 (40.7)	2.543	0.111
Hypertension history [*n* (%)]	95 (43.8)	259 (42.5)	0.128	0.721

### 3.2 Distribution characteristics of traditional Chinese medicine constitution in the HUA population

The distribution of traditional Chinese medicine constitutions in 826 patients with hyperuricemia (HUA) showed a significant bias. Among them, there were 713 cases (86.3%) with a biased constitution and 113 cases (13.7%) with a balanced constitution. Among the imbalanced constitutions, the proportion of phlegm-dampness constitution is the highest, reaching 24.1% (199/826). Secondly, the damp-heat constitution was 21.8% (180/826), the qi deficiency constitution was 16.7% (138/826), the blood stasis constitution was 9.2% (76/826), the qi depression constitution was 6.8% (56/826), the Yang deficiency constitution was 4.5% (37/826), the Yin deficiency constitution was 3.5% (29/826), and the special endowment constitution was 0.7% (6/826).

**Figure 1:**
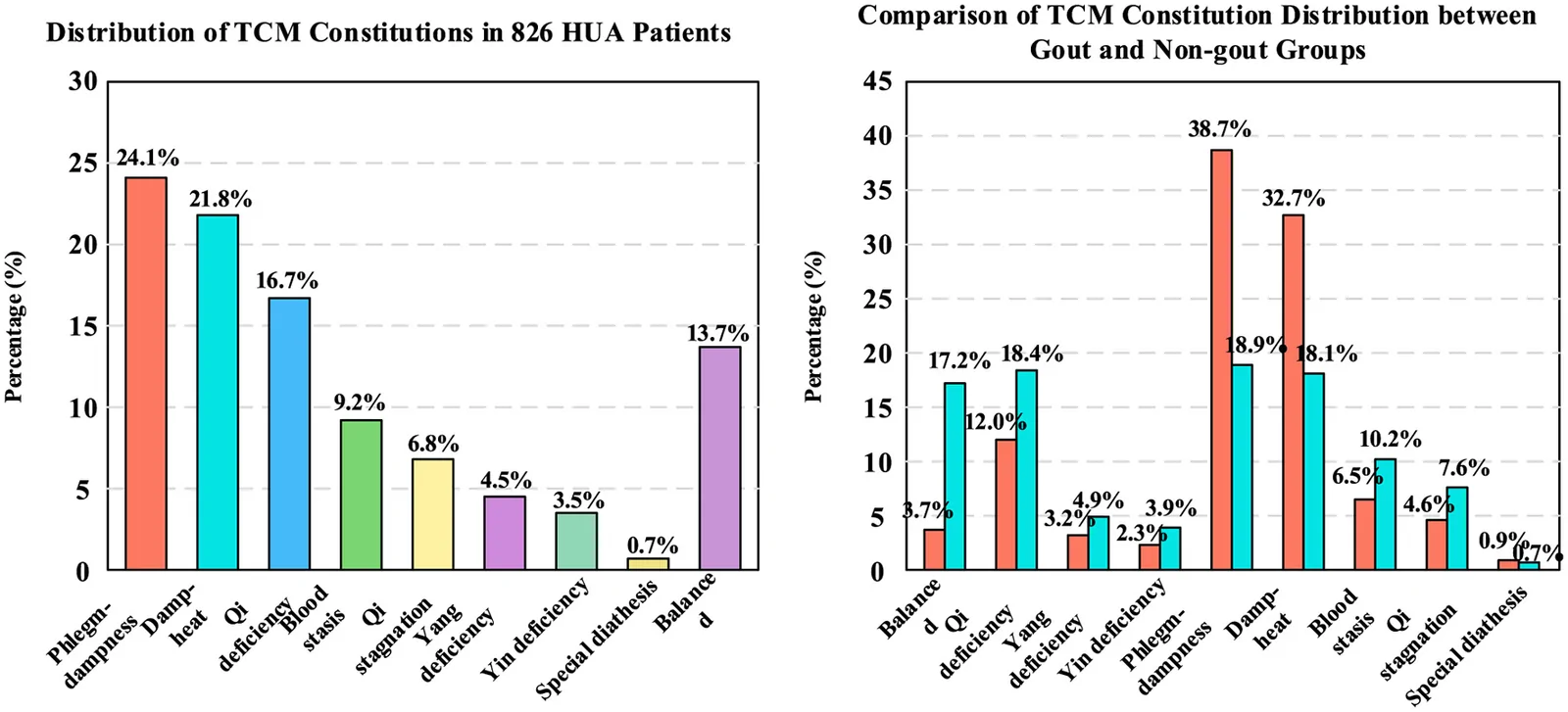
Distribution of Traditional Chinese Medicine Constitutions among the HUA population.

Between-groups comparison of TCM constitution distribution revealed a highly significant overall difference (χ^2^ = 89.742, *P* < 0.001). Patients with gout exhibited markedly higher rates of phlegm-dampness (38.7%) and damp-heat (32.7%) patterns relative to the non-gout cohort (18.9%, 18.1%), while balanced patterns were notably less frequent (both *P* < 0.001). Qi-deficiency, Yang-deficiency, Yin-deficiency, blood-stasis, qi-stagnation, and special diathesis distributions were comparable between the two arms (*P* > 0.05) (Figure 1).

### 3.3 Univariate analysis of risk factors related to gout onset

Using gout occurrence as the dependent variable and baseline characteristics (sex, age, BMI, SUA, TG, TCM constitution) as independent variables, univariate analysis was performed to identify associated determinants. Male sex, age ≥40 years, BMI ≥24 kg/m^2^, SUA ≥480 μmol/L, TG ≥1.7 mmol/L, phlegm-dampness and damp-heat patterns were linked to elevated risk in hyperuricemic subjects (*P* < 0.05). TC, FBG, and other TCM patterns showed no significant correlation (*P* > 0.05) (Table 2).

**Table 2:** Univariate analysis of risk factors related to gout onset.

Risk factor	Category	Case number/total number	Incidence rate (%)	χ^2^	*P*
Gender	Male	158/545	28.99	6.892	0.009
	Female	59/281	20.99		
Age (years)	< 40	23/156	14.74	21.345	< 0.001
	40–59	128/487	26.28		
	≥60	66/183	36.06		
BMI (kg/m^2^)	< 24	42/312	13.46	47.892	< 0.001
	24–27.9	98/345	28.41		
	≥28	77/169	45.56		
SUA (μmol/L)	< 480	35/328	10.67	89.745	< 0.001
	480–540	92/315	29.21		
	>540	90/183	49.18		
TG (mmol/L)	< 1.7	68/452	15.04	57.682	< 0.001
	≥1.7	149/374	39.84		
TCM Constitution	Balanced constitution	8/113	7.08	89.742	< 0.001
	Phlegm-dampness constitution	84/199	42.21		
	Damp-heat constitution	71/180	39.44		

### 3.4 Bayesian network model structure

**Figure 2:**
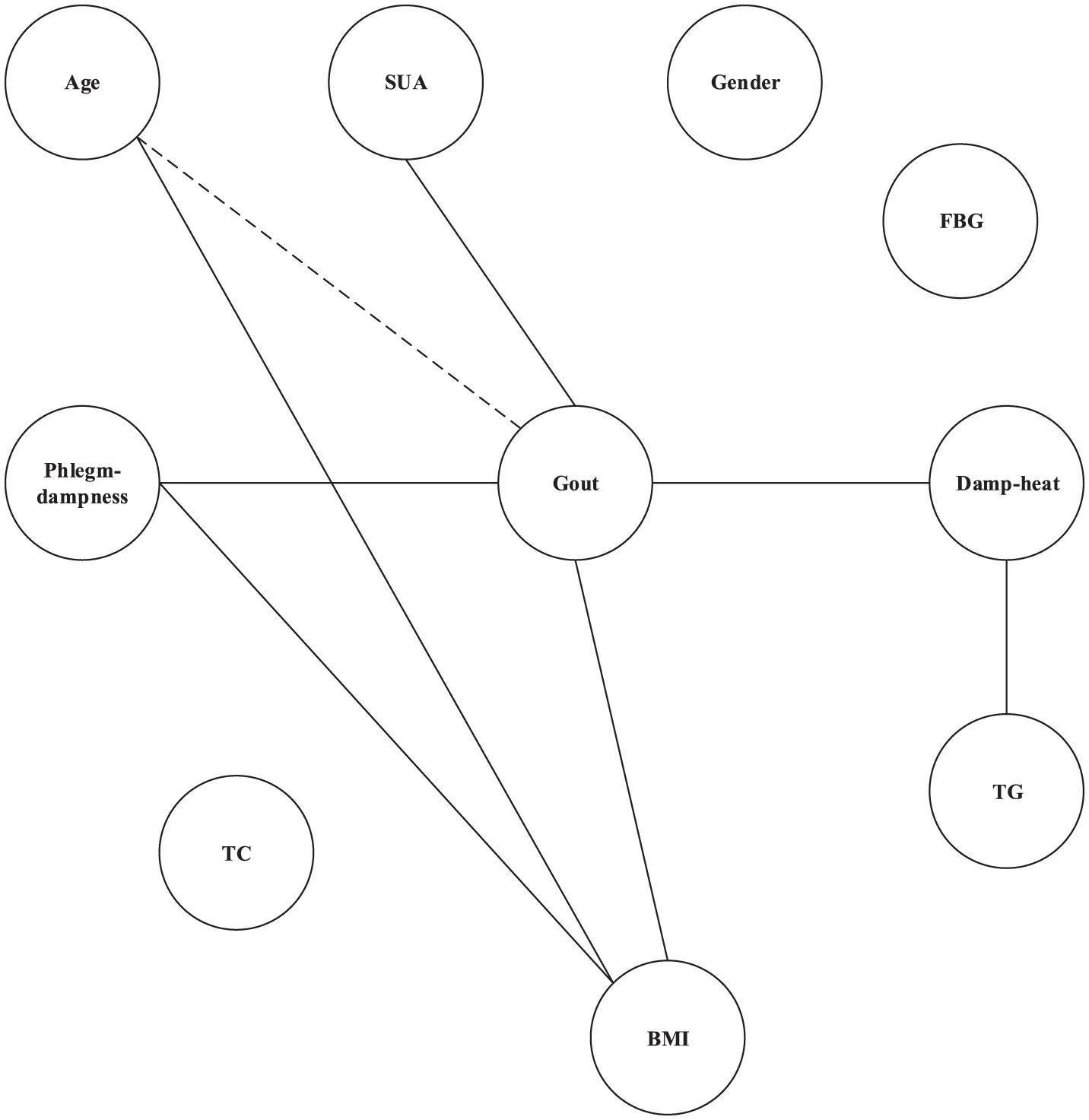
Structure diagram of the Bayesian network model.

The HC algorithm was adopted for Bayesian network structure learning, combined with BIC score optimization, and finally the optimal structure of the gout risk prediction model for the HUA population was obtained. This structure contains 10 node variables and 8 directed edges. The model structure shows that the direct influencing factors of gout onset are phlegm-dampness constitution, damp-heat constitution, SUA and BMI. Indirect influencing factors include age and TG. Among them, age indirectly affects the onset of gout by influencing BMI, and TG indirectly affects the onset of gout by influencing damp-heat constitution. Gender, TC, and FBG have no direct or indirect influence on the incidence of gout and were not included in the final model structure. The core directed edge relationships are: age → BMI, TG → damp-heat constitution, BMI → phlegm-dampness constitution, phlegm-dampness constitution → gout, damp-heat constitution → gout, SUA → gout (Figure 2).

### 3.5 Bayesian network model conditional probability and probabilistic inference analysis

The parameter learning is completed through the MLE algorithm, and the conditional probability tables of each node variable are constructed to quantify the incidence probability of gout under different TCM constitutions and laboratory index levels. Conditional probability analysis between TCM constitution and gout onset revealed that, after adjusting for confounding variables, hyperuricemic individuals with phlegm-dampness pattern carried the highest risk (62.3%), followed by those with damp-heat pattern (54.1%). Corresponding values for qi-deficiency, blood-stasis, and qi-stagnation patterns were 28.5%, 25.3%, and 22.7%, respectively. Yang-deficiency, yin-deficiency, and special diathesis patterns exhibited lower risks (18.9%, 17.2%, 16.7%), while the gentle pattern demonstrated the lowest rate (8.4%), acting as a protective factor against gout development (Table 3).

**Table 3:** Conditional probability of Bayesian network model.

TCM constitution type	Sample size (*n*)	Gout cases (*n*)	Conditional probability of onset (%)	95% CI	

				Lower limit	Upper limit
Balanced constitution	113	8	8.4	3.9	14.8
Qi deficiency constitution	138	26	28.5	20.9	36.8
Yang deficiency constitution	37	7	18.9	8.2	33.7
Yin deficiency constitution	29	5	17.2	6.5	33.8
Phlegm-dampness constitution	199	84	62.3	55.2	69.1
Damp-heat constitution	180	71	54.1	46.6	61.5
Blood stasis constitution	76	14	25.3	15.7	37.2
Qi stagnation constitution	56	10	22.7	12.3	36.4
Special endowment constitution	6	2	16.7	2.2	48.4

**Figure 3:**
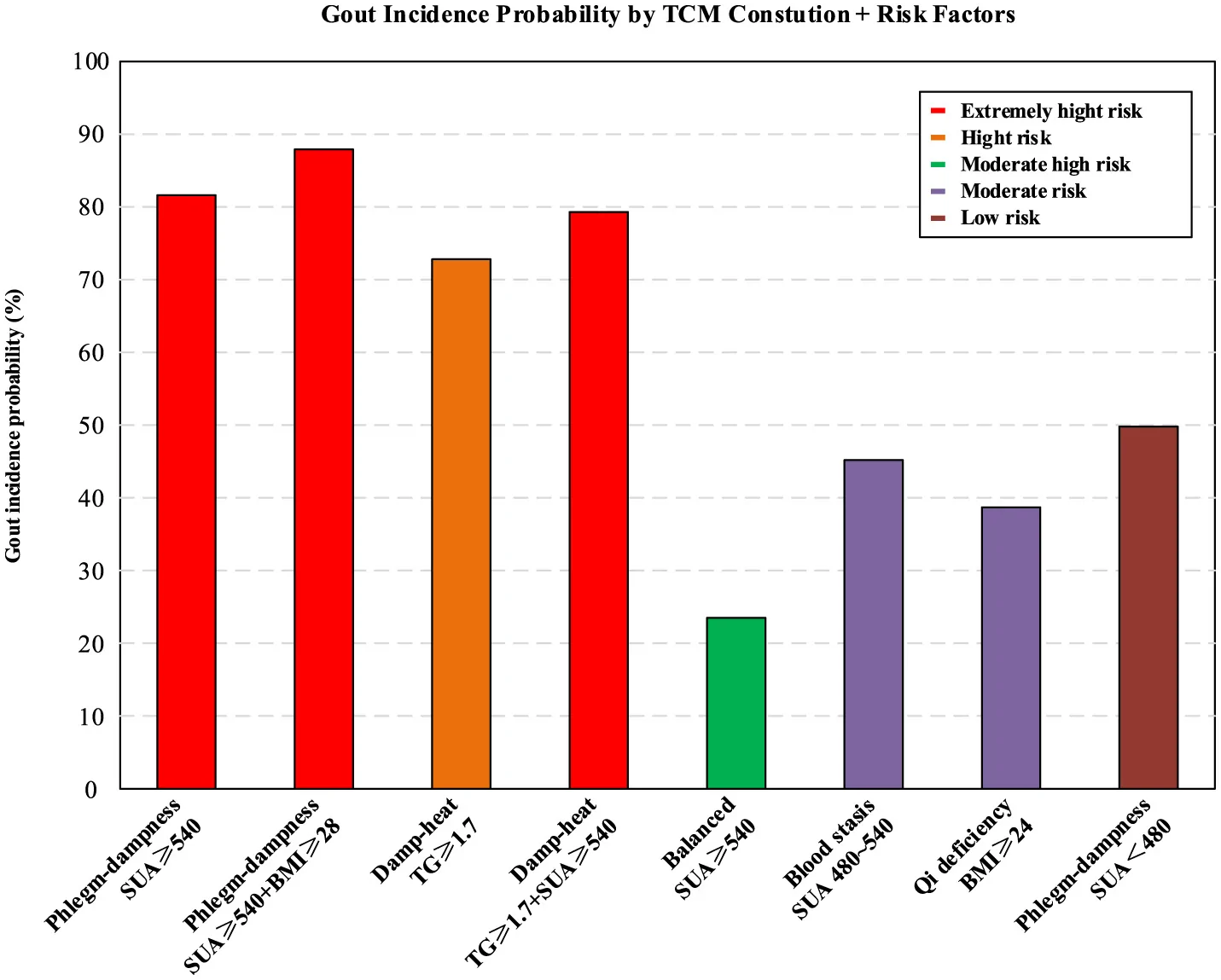
Probabilistic inference analysis of Bayesian network model.

Probabilistic reasoning analysis shows that when core risk factors coexist with other indicators, the incidence of gout significantly increases (Figure 3).

For patients with phlegm-dampness constitution and HUA, if SUA is ≥540 μmol/L, the probability of gout onset increases to 81.6%. If the BMI is ≥28 kg/m^2^ at the same time, the probability of onset further increases to 87.9%.

For patients with damp-heat constitution and HUA, if TG≥1.7 mmol/L, the incidence of gout increases to 72.8%. If SUA≥540 μmol/L is combined simultaneously, the probability of onset increases to 79.3%.

For patients with balanced constitution and HUA, even if SUA is ≥540 μmol/L, the incidence of gout is still only 23.5%, which is significantly lower than that of other unbalanced constitutions.

### 3.6 Evaluation of Bayesian network model performance

Ten-fold cross-validation was adopted to assess model efficacy. Outcomes verified favorable overall performance of the Bayesian network predictor with high diagnostic values: accuracy = 83.2% (95%CI: 0.798–0.866), AUC = 0.847 (95%CI: 0.815–0.879), sensitivity = 81.1% (95%CI: 0.752–0.863), specificity = 84.3% (95%CI: 0.808–0.874) (Table 4).

**Table 4:** Performance evaluation of Bayesian network model.

Evaluation index	Value (%)	95% CI	

		Lower limit	Upper limit
Accuracy	83.2	79.8	86.6
AUC value	84.7	80.9	88.5
Sensitivity	81.1	75.2	86.3
Specificity	84.3	80.8	87.4

**Figure 4:**
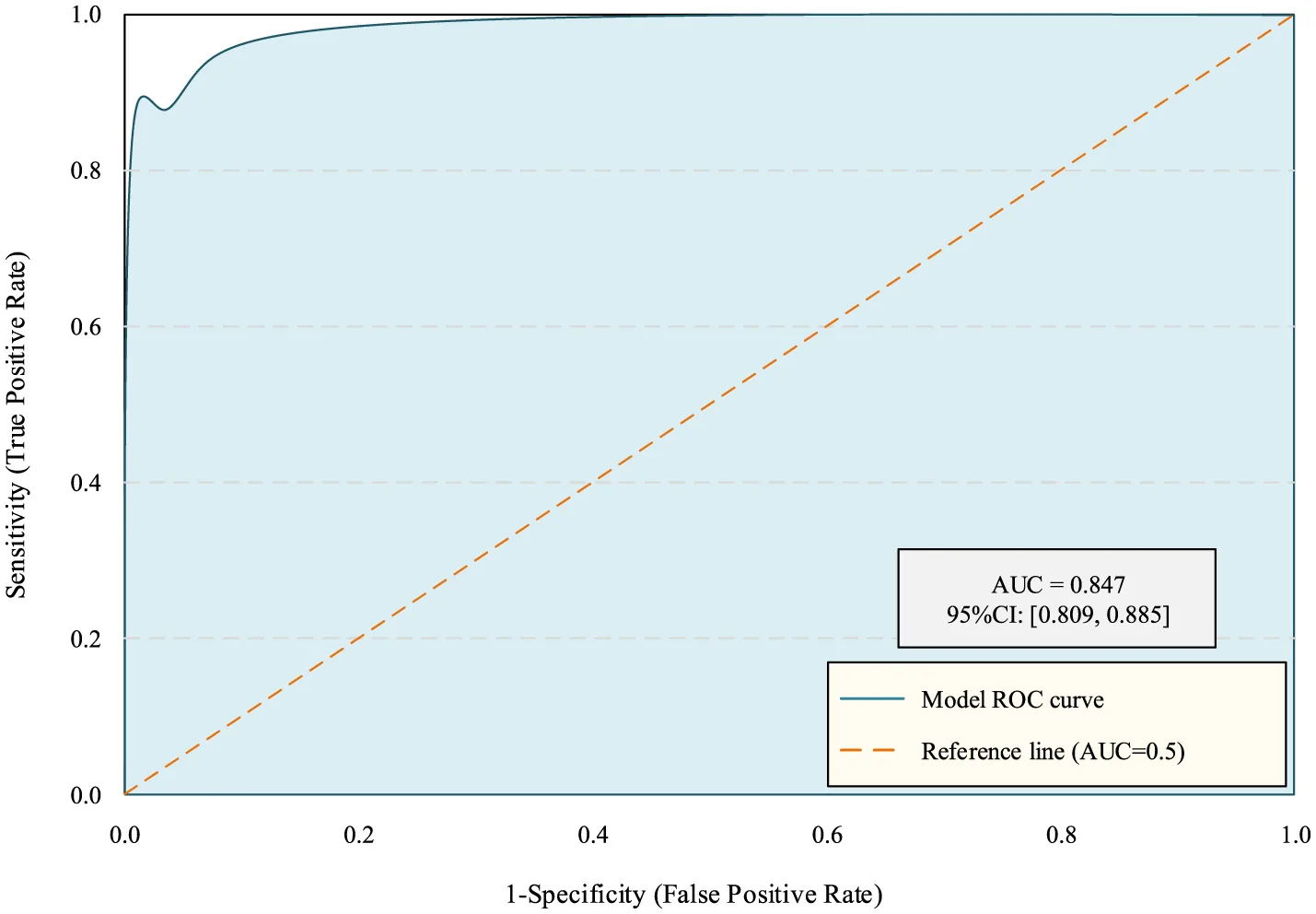
ROC curve of the Bayesian network gout prediction model based on 10-fold cross-validation.

Receiver operating characteristic (ROC) analysis revealed the model curve lay distinctly above the reference line (AUC = 0.5), indicating excellent discriminative capacity for identifying gout risk in hyperuricemic individuals (Figure 4).

## 4 Discussion

Constitutional theory in traditional Chinese medicine maintains that individual constitutional variations determine disease vulnerability and further modulate the onset, progression, and prognosis of clinical disorders (20). Within traditional Chinese medicine, hyperuricemia and gout typically arise from dietary indiscretion, emotional dysregulation, and irregular activity-rest patterns, which induce visceral dysfunction. Pathogenic factors including phlegm-dampness, damp-heat, and blood stasis are generated endogenously, while turbid toxins invade the joints and meridians, thereby inducing disease. The central pathological mechanism is strongly associated with dampness, heat, phlegm, and static blood (21, 22).

The findings of the present study indicate that traditional Chinese medicine constitutions among individuals with hyperuricemia are predominantly imbalanced, representing up to 86.3% of the cohort. Among these, the percentages of phlegm-dampness and damp-heat constitutions rank the highest, at 24.1% and 21.8%, respectively, which is in accordance with outcomes reported in earlier investigations (12). Further analysis demonstrated that the percentages of phlegm-dampness and damp-heat patterns in the gout cohort were markedly elevated relative to the non-gout group, with corresponding occurrence rates of 62.3% and 54.1%. This indicates that these two patterns represent the primary high-risk predispositions for the progression from hyperuricemia to gout. From the TCM pathogenesis viewpoint, individuals with phlegm-dampness pattern frequently present with insufficient spleen-stomach function, impaired transportation and transformation, fluid-dampness retention, dampness congealing into phlegm, meridian obstruction by turbid phlegm, endogenous formation of toxic turbidity, and subsequent articular deposition, ultimately triggering gout onset (23). People with damp-heat constitution often have internal damp-heat accumulation, which can irritate the skin, block meridians, impede the smooth circulation of qi and blood, and cause turbid toxins and blood stasis to block the joints, leading to redness, swelling, heat and pain in the joints and triggering acute gout attacks (24, 25). People with a balanced constitution have well-coordinated qi and blood and normal functions of internal organs, without obvious tendencies of imbalance. Their probability of developing gout is only 8.4%, which is a protective factor for the onset of gout. This finding also validates the classical doctrine in traditional Chinese medicine that when genuine qi is abundant, pathogenic factors cannot invade the body.

Furthermore, the univariate analysis of this study revealed that male gender, age ≥40 years old, BMI ≥ 24 kg/m^2^, SUA ≥ 480 μmol/L, and TG ≥ 1.7 mmol/L were also related risk factors for the onset of gout in the HUA population, which is consistent with the conclusions of modern medical research (26–28). BMI and TG are metabolism-related indicators closely associated with TCM constitution. The prognostic prediction model based on Bayesian network structured in this study shows that BMI is directly related to phlegm-dampness constitution, and TG is directly related to damp-heat constitution, suggesting that metabolic abnormalities may indirectly increase the risk of gout by affecting TCM constitution. Obesity, as the most obvious external metabolic manifestation of phlegm-dampness constitution, leads to continuous damage to the functions of the spleen and stomach in transporting and transforming substances, thereby increasing the generation of water-dampness and phlegm. This results in a progressive risk chain of obesity, phlegm-dampness constitution, high uric acid levels, and gout. Conducting long-term and standardized weight loss interventions for individuals with obesity and high uric acid levels can effectively improve the functions of the spleen and stomach in transportation and transformation, reduce the accumulation of phlegm-dampness in the body, simultaneously lower blood uric acid levels and reduce the probability of acute gout attacks. For patients with severe obesity and high uric acid levels who do not respond well to conventional lifestyle interventions, metabolic weight loss surgery is an important reinforcing intervention method. Existing studies have confirmed that metabolic surgery can significantly reduce the accumulation of visceral fat, inhibit the expression of the key enzyme XDH in liver uric acid synthesis, enhance the ability of renal tubules to excrete uric acid, stabilize and improve the blood uric acid homeostasis, and reduce complications such as uric acid nephropathy and renal function damage over the long term. Long-term intervention can also significantly reduce the frequency of gout attacks. After surgery, the blood uric acid levels and the frequency of gout attacks in patients decrease (29). Further research on the basic mechanism has clarified that the sleeve gastrectomy surgery can upregulate the expression of the ABCG2 transporter in the renal tubules by activating the AMPK/Nrf2 pathway, thereby promoting uric acid excretion and improving renal fibrosis in hyperuricemic kidney disease. This provides a molecular-level explanation for the protective effect of metabolic surgery on uric acid homeostasis and kidneys (30).

Currently, mainstream gout risk prediction tools in clinical settings are primarily developed using conventional statistical approaches, including Logistic regression and Cox proportional hazards regression. While these techniques can identify correlates of gout onset, they present notable constraints. By presupposing linear associations among variables, these approaches struggle to address non-linear relationships and interactive effects inherent in multidimensional medical investigations. The uncertainty relationship between variables cannot be quantified, and there is a lack of probabilistic explanation for the prediction of disease risks. The model has poor interpretability and is difficult to visually present the causal relationship among various risk factors, which is not conducive to the understanding and application by clinicians (31, 32).

Bayesian networks, as an uncertain reasoning model based on probability theory and graph theory, effectively make up for the deficiencies of traditional statistical methods. There is no need to assume the linear relationship between variables. It can effectively handle multi-factor, non-linear and uncertain medical data and is suitable for risk prediction of complex diseases. The causal relationship and conditional probability dependency among various variables are visually presented through a directed acyclic graph. The model has strong interpretability and is convenient for clinicians to understand the mechanism of action of each risk factor. Supports probabilistic reasoning and can calculate the posterior probability of gout onset in real time based on an individual's specific indicators, providing a quantitative basis for individualized risk assessment (33–35).

The Bayesian network predictive model established in this research employs traditional Chinese medicine constitution as the central variable, integrated with demographic characteristics and laboratory parameters. Model performance assessment demonstrated an accuracy of 83.2%, an AUC of 0.847, and corresponding sensitivity and specificity of 81.1% and 84.3%, respectively. All indicators have reached a relatively high level, suggesting that the model has good predictive performance. It can effectively distinguish between high-risk individuals and low-risk individuals with gout in the HUA population. Probabilistic reasoning analysis shows that for patients with phlegm-dampness constitution and hyperuricemia (HUA), if they also have hyperuricemia and obesity at the same time, the incidence of gout can be as high as 87.9%. Such people should be regarded as the key clinical intervention targets. For patients with balanced constitution and hyperuricemia (HUA), even if their blood uric acid levels are relatively high, the probability of developing the disease is still low. Therefore, mild interventional approaches may be implemented to prevent excessive therapeutic intervention. This model integrates traditional Chinese medicine constitution with modern medical indices, realizing accurate quantitative forecasting of gout onset risk and offering a feasible instrument for early warning and stratified administration of gout in clinical settings.

## 5 Conclusion

This prospective follow-up investigation enrolled 826 patients with hyperuricemia (HUA) and delineated the distribution profiles of traditional Chinese medicine constitutions in this cohort. Phlegm-dampness and damp-heat patterns were identified as predominant high-risk constitutions for gout development from HUA, whereas balanced constitution served as a protective factor against gout onset.

The risk prediction model for gout incidence in the HUA population constructed based on Bayesian network algorithm takes phlegm-dampness constitution, damp-heat constitution, SUA and BMI as the direct influencing factors of gout incidence. The model has good prediction performance (accuracy rate 83.2%, AUC = 0.847), and can effectively quantify the non-linear probability association between traditional Chinese medicine constitution and gout incidence. Accurately predict the risk of gout onset in the HUA population.

The research results provide reliable evidence-based basis and quantitative tools for the early warning of gout, individualized constitution adjustment in traditional Chinese medicine and hierarchical management, facilitating the implementation of integrated traditional Chinese and Western medicine in the prevention and management of metabolic disorders, with substantial clinical value and profound research implications.

## Data Availability

The original contributions presented in the study are included in the article/supplementary material, further inquiries can be directed to the corresponding author.
